# Life Expectancy of the Ethnically Mixed: Register-Based Evidence from Native Finns

**DOI:** 10.3390/ijerph18073415

**Published:** 2021-03-25

**Authors:** Kaarina Reini, Jan Saarela

**Affiliations:** Demography Unit, Faculty of Education and Welfare Studies, Åbo Akademi University, P.O. Box 311, FI-65101 Vaasa, Finland; jan.saarela@abo.fi

**Keywords:** life expectancy, ethnicity, mixed heritage, population registers, native groups, Finland

## Abstract

As the ethnic composition around the world is becoming more diverse, the need to produce vital statistics for ethnically mixed populations is continuously increasing. Our aim is to provide the first life expectancy estimates for individuals with uniform Finnish, uniform Swedish, and mixed Finnish-Swedish backgrounds, based on individuals in the native population of Finland who can be linked to both their parents. Life expectancy at birth in the period 2005–2015 was calculated from population and mortality numbers at the one-year level based on each person’s sex, year of birth, and the unique ethnolinguistic affiliation of the index person and each parent. Swedish-registered individuals with Swedish-registered parents had the longest life expectancy at birth, or 85.68 years (95% CI: 85.60–85.77) for females and 81.36 for males (95% CI: 81.30–81.42), as compared to 84.76 years (95% CI: 84.72–84.79) and 78.89 years (95% CI: 78.86–78.92) for Finnish-registered females and males with Finnish-registered parents. Persons with mixed backgrounds were found in between those with uniform Finnish and uniform Swedish backgrounds. An individual’s own ethnolinguistic affiliation is nevertheless more important for longevity than parental affiliation. Similar register-based analyses for other countries with mixed populations would be useful.

## 1. Introduction

Increased migration flows and rates of ethnic intermarriage have made the ethnic composition increasingly more diverse in many countries around the world. The need to produce vital statistics, and particularly reliable life tables, for ethnically mixed populations is therefore continuously increasing. The calculation of mortality rates and life expectancies for ethnic groups is challenging because measures of ethnicity are often not comparable, and particularly so for persons with a mixed ethnic origin [1,2]. The current evidence on the life expectancy of individuals with mixed ethnic backgrounds is therefore scarce. The aim of this paper is to provide the first life expectancy estimates for individuals with uniform Finnish, uniform Swedish, and mixed Finnish-Swedish backgrounds, based on individuals in the native population of Finland who can be linked to both their parents.

Finland, which is a highly developed country with modest social disparities and a long life expectancy, provides a convenient setting in which to overcome some of the major challenges of studying the life expectancy of ethnically mixed persons. The Finnish population register contains basic information about all persons residing in the country. They can be linked to their parents provided that they were born in the country, and there is complete information on births and deaths. From an international perspective, an unusual feature of the Finnish population registration system is that there are records on each person’s native language, which in practice means the unique ethnolinguistic affiliation of each person. This serves as a highly reliable measure of ethnolinguistic group membership [3]. Immigration of foreign-born individuals to Finland was very limited before 1990, and not until the past two decades has it increased in size [4]. Foreign-born immigrants are excluded from this study as most of them are young, and many cannot be linked to their parents. 

The population for which adequate life tables can be calculated therefore consists of two groups that are native and can be separated on basis of the unique ethnolinguistic affiliation: Finnish speakers and Swedish speakers. Both are guaranteed the same constitutional rights and are equal in most observable respects. There are, for instance, parallel education systems for Finnish speakers and Swedish speakers from kindergarten up to the university level, separate parishes, and services in the public, private, and third sectors that serve each group. Both groups, therefore, have access to similar services of the same quality. 

Not until the 20th century, and particularly during its latter part, did intermarriage and births in mixed Finnish-Swedish unions became more common. By observing the ethnolinguistic affiliation of the mother and the father, we can therefore know who comes from a mixed family. Currently, 88% of the total population consists of persons registered as Finnish speakers, and 5% of persons are registered as Swedish speakers [5]. During the past decades, births within mixed unions have been equally as frequent as births within endogamous Swedish-speaking unions. There is consequently a considerable degree of ethnolinguistic mix within the contemporary population for which life expectancy tables can be calculated.

The registration of ethnolinguistic affiliation usually occurs at birth or soon thereafter and is made by the parents. Within non-mixed unions, the choice of ethnolinguistic affiliation for the child is straightforward. If both parents have the same affiliation, the child will also be registered in that same way. Within mixed families, registration is more of an issue and based on a choice made by the parents [6]. The choice has few, if any, binding and immediate consequences. In most cases, it is still a strong indicator of what will be the dominant culture throughout childhood and young adulthood, that is, which of the two parallel educational tracks is taken and whether a person grows up in the Finnish-speaking or the Swedish-speaking local community.

Based on this setting with multigenerational population register data in which each person’s and the parents’ ethnolinguistic affiliation can be identified, the aim of this paper is to provide the first life expectancy estimates for individuals with uniform Finnish, uniform Swedish, and mixed Finnish-Swedish backgrounds. This is accomplished by calculating life expectancy at birth in the period 2005–2015 from the population and mortality numbers at the one-year level based on each person’s sex, year of birth, and the unique ethnolinguistic affiliation of the index person and his or her mother and father. 

## 2. Materials and Methods

### 2.1. Data

The data used contained all individuals who lived in Finland at the beginning of each calendar year in the period 1971–2015. All deaths during each calendar year are known. Each index person can be linked to his or her mother and father, provided that the parent had not died before the end of 1970. Biological and adopted children can be separated, but since the latter group is small, we make no distinction. For persons born before 1970, an additional requirement for linkage is that the child lived in the same household as the parent. For all cohorts born after 1952, that is, persons aged under 18 years in 1970, child–parent links can therefore be established with great precision, while there are more missing links for earlier born cohorts. 

Each person’s sex, birth year, year of death, and ethnolinguistic affiliation (native language, or mother tongue) are known with full precision. Because of the multigenerational structure, meaning that we can link children to their parents, the ethnolinguistic affiliation of each parent is also known. We can therefore separate individuals according to their own, the mother’s, and the father’s ethnolinguistic affiliation. All analyses concern Finnish- and Swedish-registered persons with Finnish- or Swedish-registered mothers and fathers. Foreign-born immigrants are thus beyond the scope of this paper. They cannot generally be linked to their parents unless they had lived with their parents in Finland. Immigration of foreign-born individuals was very limited before 1990, and not until the past two decades has it increased in size. Most of the foreign-born immigrants, with ethnicities other than Finnish or Swedish, are also young. Life tables of the type that we constructed here for Finnish and Swedish speakers cannot, therefore, be adequately calculated for other ethnic groups.

It is possible to change ethnolinguistic affiliation, but few persons do so [6]. Here, we consider a person to be Swedish-registered if he or she has ever been Swedish-registered. Switching the typology to mean Finnish-registered if the person has ever been Finnish-registered does not change the results to any noteworthy degree.

The data are register-based and anonymized. All data access, data preparation, and analyses were performed within Statistics Finland’s remote access system, Fiona [7]. Our contract number is TK-52–694-18. Our use of Fiona following Statistics Finland’s guidelines for handling data [8] implied that there was no need to seek separate ethical approval for this study alone. The data can be obtained from Statistics Finland by other researchers, provided that a research application is approved and service fees are paid.

### 2.2. Statistical Analysis

Life expectancy at birth in the period 2005–2015 was calculated from population and mortality numbers at the one-year level based on each person’s sex, year of birth, and the unique ethnolinguistic affiliation of the index person and each parent. One-year intervals were used to obtain data series that had a relatively large number of data points over calendar time. The study period ends in 2015 because, in the data used, the last cohort we can link to the parents consists of persons born in 2015. The study period starts in 2005 because the calculations of life expectancy are based on cohorts for whom we can link most of the parents. For cohorts born before 1945, the linkage is largely missing. For these cohorts, we needed to impute the mortality rates after age 60, and we did so based on each person’s ethnolinguistic affiliation alone, that is, without the mother’s and the father’s ethnolinguistic affiliation. The R package Demography was used to compile the life tables [9,10].

## 3. Results

In 2015, life expectancy at birth was 79.09 years (95% CI: 79.06–79.12) for Finnish-registered men, 80.99 years (95% CI: 80.92–81.06) for Swedish-registered men, 84.88 years (95% CI: 84.85–84.91) for Finnish-registered women, and 85.50 years (95% CI: 85.43–85.57) for Swedish-registered women (Table 1). Thus, the life expectancy of Finnish-registered men is expected to be 1.90 years shorter than that of Swedish-registered men, while the difference in women is 0.62 years. These estimates are almost identical to recent calculations of Statistics Finland [11] and in line with previous research [12,13].

When applying the imputation, life expectancy at birth is 79.18 years (95% CI: 79.15–79.21) for Finnish-registered men, 81.27 years (95% CI: 81.20–81.34) for Swedish-registered men, 84.93 years (95% CI: 84.90–84.96) for Finnish-registered women, and 85.54 years (95% CI: 85.47–85.61) for Swedish-registered women. Thus, only for Swedish-registered men does the imputation affect the estimate to any notable degree.

The difference between Finnish- and Swedish-registered persons is larger when individuals with uniform backgrounds are being compared, or 2.47 years in men and 0.92 years in women. Life expectancy at birth is 78.89 (95% CI: 78.86–78.92) years for Finnish-registered men with both parents Finnish, 81.36 years (95% CI: 81.30–81.42) for Swedish-registered men with both parents Swedish, 84.76 years (95% CI: 84.72–84.79) for Finnish-registered women with both parents Finnish, and 85.68 years (95% CI: 85.60–85.77) for Swedish-registered women with both parents Swedish. Persons with mixed origins, that is, one Finnish-registered parent and one Swedish-registered parent, lie between the uniform Finnish and uniform Swedish groups. An individual’s own affiliation is nevertheless more important than parental affiliation. Finnish-registered men with mixed backgrounds have a life expectancy at birth of 79.25 years (95% CI: 79.17–79.33), which is 0.36 years longer than that of Finnish-registered men with both parents Finnish-registered. Swedish-registered men with mixed background have a life expectancy at birth of 81.34 years (95% CI: 81.26–81.42), which is 0.02 years shorter than that of Swedish-registered men with both parents Swedish-registered. For women, these differences are somewhat higher. Finnish-registered women with mixed backgrounds have a life expectancy at birth of 85.15 years (95% CI: 85.06–85.24), which is 0.39 years longer than that of Finnish-registered women with both parents Finnish-registered. Swedish-registered women with mixed backgrounds have a life expectancy at birth of 85.32 years (95% CI: 85.23–85.40), which is 0.36 years shorter than that of Swedish-registered women with both parents Swedish-registered. For persons with mixed backgrounds, the difference at birth between Finnish-registered and Swedish-registered persons is consequently 2.09 years (79.25–81.34) for male life expectancy and 0.17 years for female life expectancy (85.15–85.32). Thus, in women, mixed backgrounds relate to a smaller difference in life expectancy at birth (0.17 vs. 0.61), while for men, it does not (2.09 vs. 2.09).

**Figure 1:**
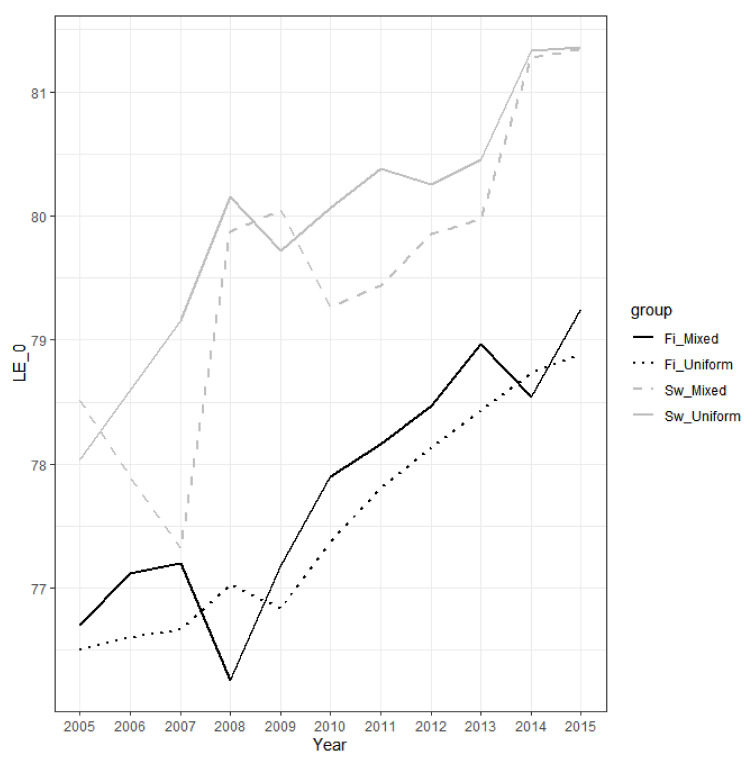
Male life expectancy at birth in the period 2005–2015 by an individual’s own and parental ethnolinguistic affiliation. Notes: ‘Fi_Mixed’ is Finnish-registered with mixed backgrounds, ‘Fi_Uniform’ is Finnish-registered with uniform Finnish backgrounds, ‘Sw_Mixed’ is Swedish-registered with mixed backgrounds, and ‘Sw_Uniform’ is Swedish-registered with uniform Swedish backgrounds. The estimates with 95% CIs are found in Table S1 in the Supplementary Materials. LE_0 refers to life expectancy at birth.

**Figure 2:**
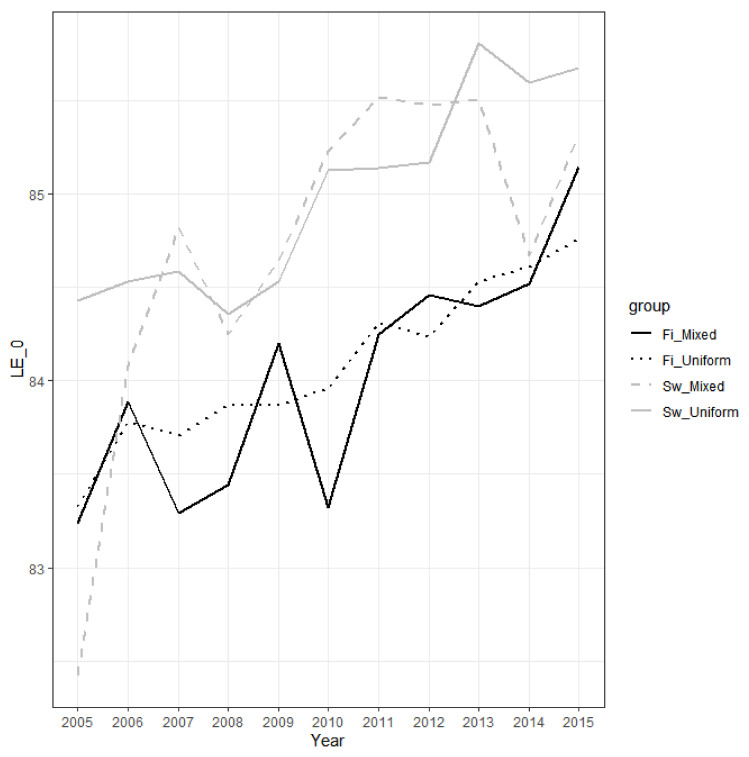
Female life expectancy at birth in the period 2005–2015 by own and parental ethnolinguistic affiliation. Notes: ‘Fi_Mixed’ is Finnish-registered with mixed backgrounds, ‘Fi_Uniform’ is Finnish-registered with uniform Finnish backgrounds, ‘Sw_Mixed’ is Swedish-registered with mixed backgrounds, and ‘Sw_Uniform’ is Swedish-registered with uniform Swedish backgrounds. The estimates with 95% CIs are found in Table S2 in the Supplementary Materials. LE_0 refers to life expectancy at birth.

The between-group differences in life expectancy at birth are fairly stable over the study period (2005–2015): (Figure 1 and Figure 2). It increased by 3.3 years, from 78.03 to 81.36, for Swedish-registered men with both parents Swedish-registered, while the increase was 2.4 years, from 76.50 years to 78.89 years, for Finnish-registered men with both parents Finnish-registered. In women, the corresponding increases were 1.2 years (84.43–85.68) and 1.4 years (83.33–84.76). For the smaller subgroups, there is more variation, but the overall pattern supports the notion that the differences in life expectancy by ethnolinguistic affiliation are fairly stable over time, with some tendencies towards a widening in men and a narrowing in women. Previous researchers using data from earlier decades, based on one-generation ethnolinguistic affiliation only, have reached similar conclusions [11].

## 4. Discussion

Ethnicity, and mixed ethnicity in particular, is a complex and multidimensional concept that introduces methodological challenges for the study of life expectancy and its variation across population subgroups. It relates to various elements, such as individual self-assessment, ancestry, national identity, religion, and country of birth [1,14]. In comparison with measures like occupation and education, for instance, there exists no standard or widely accepted measurement protocol [2]. Although self-reported ethnicity has been considered the most accurate measure of ethnicity, it has limitations [14,15]. Persons with mixed backgrounds may identify themselves according to the ethnicity of either parent or as having a mixed ethnicity [16]. Surveys tend to assume stability in the meaning of what an ethnic group is, and responses may be affected by the nature and wording of the questions and categories available [17]. If these questions evolve between surveys, the classification may become more complex over time, and the measurement may become increasingly more problematic [1]. Self-assessed ethnicity is also sensitive to how ethnicity is perceived, and the perception may change over time and across generations [16,17]. Another approach to measuring ethnicity is to use information about the country of birth [18,19]. It does not capture second and later generations, descendants of immigrants, or mixed ethnic backgrounds and is therefore useful only for recent migrant groups. Combinations of methods have also been used, such as self-reported ethnicity together with the country of birth [20]. Other approaches have been to measure ethnicity through indirect methods, such as the ecological or geocoding approach [21], the standardized illness ratio [22], and the geographically weighted method [23]. These techniques often result in complex models for obtaining mortality rates and may lead to misclassification of ethnic minority groups in particular.

In this paper, the concept of ethnicity is register-based and relates to how a person and his or her parents are ethnolinguistically classified in the Finnish population register. This taxonomy fits well with the notion that parentage is important in determining ethnic identity [24] and that the contribution of parental ethnicity to ethnic identity is more salient than social factors, such as the wider society’s perceptions, feelings of group allegiance, or identification by friends and peers [25]. 

The difference in life expectancy between Swedish-registered and Finnish-registered persons in Finland is well documented [11,12,13], but the ethnolinguistic affiliation of the parents has not been previously utilized to estimate life tables, as we have done here. We have consequently calculated life tables for native persons with mixed and non-mixed heritage, representing a demographically stable population with practically no intermarriage across other ethnic boundaries. 

We find that Swedish heritage is associated with longer life expectancy at birth than Finnish heritage. Swedish-registered men with both parents Swedish-registered are expected to live 2.5 years longer than Finnish-registered men with both parents Finnish-registered, while the corresponding difference in women is 0.9 years. Persons with mixed backgrounds are found in between those with uniform Finnish and uniform Swedish backgrounds.

Like in many other countries, education and income are also important determinants of life expectancy in Finland [26,27]. Decomposition analyses lie beyond the scope of this paper. We cannot, therefore, say how much of the difference in life expectancy between the groups relates to education, income, and other socioeconomic factors. Previous research concerned with age-specific mortality rates found that only one-tenth of the difference between Finnish- and Swedish-registered persons in Finland could be attributed to standard socioeconomic and demographic factors [12,28]. The reason is presumably that both groups are native and equally positioned in society, with similar constitutional rights. The finding is in contrast with the situation in many other countries. In the United States, for instance, socioeconomic differences account for three-quarters of the black-white gap in life expectancy [29,30]. Thus, there are reasons to believe that socioeconomic and geographic inequalities likely play a modest role in the differences in life expectancy at birth between the groups studied here, although the issue is certainly an avenue for future research.

Based on our comparison of groups that differ by their own and parental ethnolinguistic affiliation, we believe that culturally related factors may be important for the differences observed. An individual’s own affiliation is also found to be more important for life expectancy than parental affiliation. Swedish-registered persons with mixed backgrounds have a life expectancy at birth that is close to, but shorter than, Swedish-registered persons with uniform Swedish backgrounds, while Finnish-registered persons with mixed backgrounds have a life expectancy at birth that is close to, but longer than, Finnish-registered persons with uniform Finnish backgrounds. An individual’s own affiliation largely reflects whether a person has been raised within the Swedish-speaking or Finnish-speaking local community. The differences observed may therefore potentially reflect group-specific cultural practices [15,31] and the fact that marital stability and social integration are higher in the Swedish-speaking than in the Finnish-speaking community [32], and patterns of unhealthy alcohol consumption are less visible [13,33]. However, we can only speculate about the relevance of such factors.

Apart from the role of any confounding variables, which we admittedly have not addressed directly, selection or attrition may be listed as other concerns. Considering that we used data on the complete population that covers a period of 45 years, we do not believe that these are serious problems. All deaths are recorded in the population register, and attrition may therefore occur due to emigration; in order to have an effect on our findings, it would mean that the study groups must differ in health-selected migration abroad. Another selection issue concerns the measure of ethnolinguistic registration if healthier people register into a specific group. However, it is not common, and particularly not so among persons with uniform backgrounds, to change the ethnolinguistic affiliation in the population register. An issue of potentially more relevance, on the other hand, is that an individual’s own and parental ethnolinguistic affiliation may not be sufficient to identify a person’s heritage with great precision, because some of the parents have mixed Finnish-Swedish heritage.

The most important restriction of our study is that Finnish population register data cannot link persons in early-born cohorts to their parents. For cohorts born before 1945, we therefore had to impute the mortality rates after age 60 based on each person’s ethnolinguistic affiliation alone, i.e., without knowing the mother’s and the father’s ethnolinguistic affiliation. Within a few decades, studies similar to ours will not suffer from this problem, because basically all persons in the native-born population in Finland can then be linked to their parents. Like with many other ethnically mixed groups [34], the ones that were studied here have emerged quite recently. For this reason, it is necessary to repeat our analyses in future decades and to undertake similar studies for the offspring of mixed unions in other countries when these population subgroups age and grow in size. 

## 5. Conclusions

We provide the first life expectancy estimates for individuals with uniform and mixed ethnolinguistic backgrounds, based on individuals in the native population of Finland who can be linked to both their parents. Swedish-registered individuals with Swedish-registered parents are found to have the longest life expectancy at birth, as compared to Finnish-registered individuals with Finnish-registered parents. Persons with mixed backgrounds are placed in between those with uniform Finnish and uniform Swedish backgrounds. An individual’s own ethnolinguistic affiliation is nevertheless more important for longevity than parental affiliation. Similar register-based analyses for other countries with mixed populations would be useful.

## Figures and Tables

**Table 1:** Life expectancy at birth in Finland in 2015 by individuals’ own and parental ethnolinguistic affiliation for men and women.

	Men			Women		
	LE(0)	95% CI	N	LE(0)	95% CI	N
Finnish-registered individuals	79.09	(79.06–79.12)	2,406,909	84.88	(84.85–84.91)	2,498,754
Swedish-registered individuals	80.99	(80.92–81.06)	153,115	85.50	(85.43–85.57)	151,718
Finnish-registered, using imputation	79.18	(79.15–79.21)	1,972,240	84.93	(84.90–84.96)	1,886,279
Swedish-registered, using imputation	81.27	(81.20–81.34)	119,090	85.54	(85.47–85.61)	108,439
Finnish-registered with uniform Finnish backgrounds	78.89	(78.86–78.92)	1,937,330	84.76	(84.72–84.79)	1,853,845
Swedish-registered with uniform Swedish backgrounds	81.36	(81.30–81.42)	86,765	85.68	(85.60–85.77)	77,407
Finnish-registered with mixed backgrounds	79.25	(79.17–79.33)	34,910	85.15	(85.06–85.24)	32,434
Swedish-registered with mixed backgrounds	81.34	(81.26–81.42)	32,325	85.32	(85.23–85.40)	31,032

For all rows except the first two, N refers to the number of individuals for whom we can link both parents. LE(0) refers to life expectancy at birth.

## Data Availability

The data are register-based and anonymized. All data access, data preparation, and analyses were performed within Statistics Finland’s remote access system Fiona. Our contract number is TK-52-694-18. The data can consequently be obtained from Statistics Finland by other researchers, provided that a research application is approved and service fees are paid.

## Supplementary Materials

The following are available online at https://www.mdpi.com/1660-4601/18/7/3415/s1: Table S1: Estimates for male life expectancy at birth displayed in Figure 1 with 95% confidence intervals; Table S2: Estimates for female life expectancy at birth displayed in Figure 2 with 95% confidence intervals.
