# Supplementary material for: Life Expectancy of the Ethnically Mixed: Register-Based Evidence from Native Finns
Source: Int J Environ Res Public Health. 2021 Mar 25;18(7):3415. doi: 10.3390/ijerph18073415 (PMC8037163; doi:10.3390/ijerph18073415)
Supplement: Supplementary file 1 [file ijerph-18-03415-s001.pdf]

Table S1. Estimates for male life expectancy at birth displayed in Figure 1 with 95% confidence intervals

	Finnish-registered with uniform Finnish background		Swedish-registered with uniform Swedish background		Finnish-registered with mixed background		Swedish-registered with mixed background	
Year	LE(0)	95% CI	LE(0)	95% CI	LE(0)	95% CI	LE(0)	95% CI
2005	76.51	(76.47-76.55)	78.04	(77.95-78.13)	76,70	(76.54-76.86)	78.51	(78.43-78.59)
2006	76,60	(76.56-76.64)	78,60	(78.52-78.68)	77.12	(77.04-77.20)	77,90	(77.82-77.98)
2007	76.67	(76.64-76.70)	79.16	(79.10-79.22)	77,20	(77.12-77.28)	77.32	(77.24-77.40)
2008	77.04	(77.00-77.08)	80.14	(80.08-80.20)	76.26	(76.17-76.35)	79.87	(79.79-79.95)
2009	76.84	(76.81-76.87)	79.73	(79.65-79.81)	77.18	(77.10-77.26)	80.05	(79.97-80.13)
2010	77.37	(77.33-77.41)	80.06	(80.00-80.12)	77,90	(77.82-77.98)	79.27	(79.19-79.35)
2011	77.81	(77.77-77.85)	80.38	(80.32-80.44)	78.16	(78.07-78.25)	79.44	(79.36-79.52)
2012	78.13	(78.09-78.17)	80.25	(80.19-80.31)	78.47	(78.38-78.56)	79.86	(79.78-79.94)
2013	78.43	(78.40-78.46)	80.45	(80.39-80.51)	78.97	(78.88-79.06)	79.97	(79.89-80.05)
2014	78.73	(78.70-78.76)	81.33	(81.27-81.39)	78.54	(78.45-78.63)	81.28	(81.20-81.36)
2015	78.89	(78.86-78.92)	81.36	(81.30-81.42)	79.25	(79.17-79.33)	81.34	(81.26-81.42)

LE(0) refers to life expectancy at birth.

Table S2. Estimates for female life expectancy at birth displayed in Figure 2 with 95% confidence intervals

	Finnish-registered with uniform Finnish background		Swedish-registered with uniform Swedish background		Finnish-registered with mixed background		Swedish-registered with mixed background	
Year	LE(0)	95% CI	LE(0)	95% CI	LE(0)	95% CI	LE(0)	95% CI
2005	83.33	(83.30-83.36)	84.43	(84.37-84.49)	83.42	(83.33-83.51)	82.42	(82.34-82.50)
2006	83.78	(83.75-83.81)	84.53	(84.44-84.62)	83.89	(83.80-83.98)	84.08	(83.98-84.18)
2007	83.71	(83.67-83.75)	84.59	(84.53-84.65)	83.29	(83.20-83.38)	84.82	(84.73-84.91)
2008	83.87	(83.84-83.90)	84.36	(84.27-84.45)	83.44	(83.35-83.53)	84.25	(84.17-84.33)
2009	83.87	(83.83-83.91)	84.53	(84.42-84.64)	84,20	(84.11-84.29)	84.65	(84.57-84.73)
2010	83.96	(83.92-84.00)	85.13	(85.07-85.19)	83.32	(83.22-83.42)	85.23	(85.15-85.31)
2011	84.31	(84.27-84.35)	85.14	(85.08-85.20)	84.25	(84.16-84.34)	85.52	(85.38-85.66)
2012	84.24	(84.21-84.27)	85.17	(85.11-85.23)	84.46	(84.34-84.58)	85.48	(85.40-85.56)
2013	84.53	(84.50-84.56)	85.81	(85.75-85.87)	84,40	(84.31-84.49)	85.51	(85.43-85.59)
2014	84.61	(84.58-84.64)	85,60	(85.54-85.66)	84.52	(84.43-84.61)	84.67	(84.59-84.75)
2015	84.76	(84.73-84.79)	85.68	(85.59-85.77)	85.15	(85.06-85.24)	85.32	(85.24-85.40)

LE(0) refers to life expectancy at birth.
